# Supplementary material for: Exploring Peptaibol’s Profile, Antifungal, and Antitumor Activity of Emericellipsin A of Emericellopsis Species from Soda and Saline Soils
Source: Molecules. 2022 Mar 7;27(5):1736. doi: 10.3390/molecules27051736 (PMC8911692; doi:10.3390/molecules27051736)
Supplement: Supplementary file 1 [file molecules-27-01736-s001.zip › molecules-1617286-supplementary.pdf]

Supplementary

Table S1. List of isolates used in this study

Species	No. of Strains, No. of VKM, VKPM, CBS Collections	GenBank acessions
Emericellopsis alkalina Bilanenko & Georgieva	E101, F-4108, CBS 127350 T	KC987171
	A118, VKPM F1428	KC987155
	M20, FW-3040, CBS 120044	KC987169
	A103	KC987140
	A112	KC987149
	A113, FW-1476	KC987150
	A114, FW-1473	KC987151
	A115, FW-1474	KC987152
	A116	KC987153
	A117, FW-1471	KC987154
	A119	KC987156
	A120	KC987157
	A121	KC987158
	A122	KC987159
	A123	KC987160
	A124	KC987161
	A125	KC987162
	A126	KC987163
	A127	KC987164
	A128	KC987165
	M14, F-3905, CBS 120043	KC987168
	M71, F-3907, CBS 120049	KC987170
	6KS10-2, SLF 0110.0602	OM489386
	3KS17-13, SLF 0117.0313	OM489387
	5KS17-3, SLF 0117.0503	OM489388
	9KS17-3, SLF 0117.0903	OM489389
	6KS17-1, SLF 0117.0601	OM489390
	7KS17-1, SLF 0117.0701	OM489391
	8KS17-1, SLF 0117.0801	OM489392
	10KS17-1, SLF 0117.1001	OM489393
	14KS17-1, SLF 0117.1401	OM489394
	1KS18-5, SLF 0118.0105	OM489395
Emericellopsis cf. *maritima* Beliakova	1KS17-1, SLF 0117.0101	OM489381
	3KS17-1, SLF 0117.0301	OM489384
Emericellopsis cf. *terricola* J.F.H. Beyma	1KS17-4, SLF 0117.0104	OM489382
Emericellopsis sp.	2KS17-1, SLF 0117.0201	OM489383
Emericellopsis sp.	3KS17-2, SLF 0117.0302	OM489385
Emericellopsis sp.	3KS17-12, SLF 0117.0312	OM489380

Table S2. Sequences retrieved from GenBank [50] for phylogenetic analysis

Species	Acession number	GenBank acessions
Emericellopsis atlantica L.W. Hou, Crous, Rämä & Hagestad	TS7 / CBS 147198T	OL539742.1
Emericellopsis cladophorae M. Gonçalves, T. Vicente & A. Alves	MUM 19.33 / CMG25T	MK986711
Emericellopsis donezkii Beliakova	CBS 489.71T	NR_156195.1
Emericellopsis enteromorphae M. Gonçalves, T. Vicente & A. Alves	MUM 19.34 / CMG26T	MK986712
Emericellopsis glabra (J.F.H. Beyma) Backus & Orpurt	CBS 119.40T	NR_145024.1
Emericellopsis humicola (Cain) Cain ex Grosklags & Swift	CBS 180.56T	NR_145025.1
Emericellopsis koreana Hyang B. Lee, S.J. Jeon & T.T.T. Nguyen	CNUFC-MOG1-1T	MH173304
Emericellopsis maritima Beliakova	CBS 491.71T	KC987175
Emericellopsis microspora Backus & Orpurt	CBS 380.62T	NR_156196.1
Emericellopsis minima Stolk	CBS 190.55T	KC987173
Emericellopsis mirabilis (Malan) Stolk	CBS 177.53T	AY632656
Emericellopsis pallida Beliakova	CBS 490.71T	KC987176
Emericellopsis persica Papizadeh, Wijayaw., Soudi & K.D. Hyde	IBRC-M 30046T	KX668543
Emericellopsis phycophila M. Gonçalves, T. Vicente & A. Alves	MUM 19.32 / CMG15T	MK986701
Emericellopsis pusilla P.N. Mathur, Sukapure & Thirum.	CBS 226.62T	MH858143
Emericellopsis robusta Emden & W. Gams	CBS 489.73	AY632664
Emericellopsis robusta Emden & W. Gams	CBS 105.70	MH859510
Emericellopsis salmosynnemata Grosklags & Swift	CBS 382.62	AY632666
Emericellopsis stolkiae D.E. Davidson & M. Chr.	CBS 159.71T	NR_156197.1
Emericellopsis synnematicola P.N. Mathur & Thirum.	CBS 176.60T	AY632665.1
Emericellopsis terricola J.F.H. Beyma	CBS 120.40T	U57676
Emericellopsis terricola J.F.H. Beyma	CBS 229.59	AY632662
Acremonium sclerotigenum (Moreau & R. Moreau ex Valenta) W. Gams	CBS 124.42T	MH856101
Acremonium sp.	A109	KC987146

Table S3. Antimicrobial activity of the studied strains

№	No. of Strains	Zone, mm			
		Extract from culture fluid		Mycelium extract	
		A.niger ATCC 16404	*C.albicans* ATCC 2091	*A.niger* ATCC 16404	*C.albicans* ATCC 2091
		Soda soils			
1	E101 T	17	15	0	0
2	A112	15	10	0	0
3	A113	10	20	7	7
4	A120	19	10	10	10
5	A121	11	13	11	0
6	A123	19	12	17	10
7	A124	10	11	23	0
8	A125	8	10	20	0
9	A126	9	11	15	0
10	A127	0	10	0	0
11	M14	0	9	0	0
12	5KS17-3	21	21	9	0
13	6KS17-1	14	28	0	0
14	7KS17-1	21	12	9	9
15	8KS17-1	16	28	0	0
16	9KS17-3	17	15	9	9
17	10KS17-1	23	21	0	0
18	14KS17-1	17	18	9	0
19	6KS10-2	11	14	9	9
		Soda-chloride-sulfate soils			
1	A103	15	0	0	0
2	A116	10	19	8	0
		Chloride soils			
1	A114	16	-	0	0
2	A122	10	14	9	0
3	3KS17-13	19	21	9	0
4	1KS17-1	26	13	0	0
5	1KS17-4	0	0	0	0
6	2KS17-1	10	12	9	9
7	3KS17-1	0	0	0	0
8	3KS17-2	0	0	0	0
9	3KS17-12	0	0	0	0
		Chloride-sulfate soils			
1	A115	9	0	8	0
2	A117	21	21	8	9
3	A119	15	12	10	0
		Soda-chloride soils			
1	A118	26	28	10	10

2	M20	26	20	10	10
			Sulfate-soda soils		
1	A128	10	9	9	-
2	M71	9	9	0	9
			Undefined		
1	1KS18-5	12	16	9	9

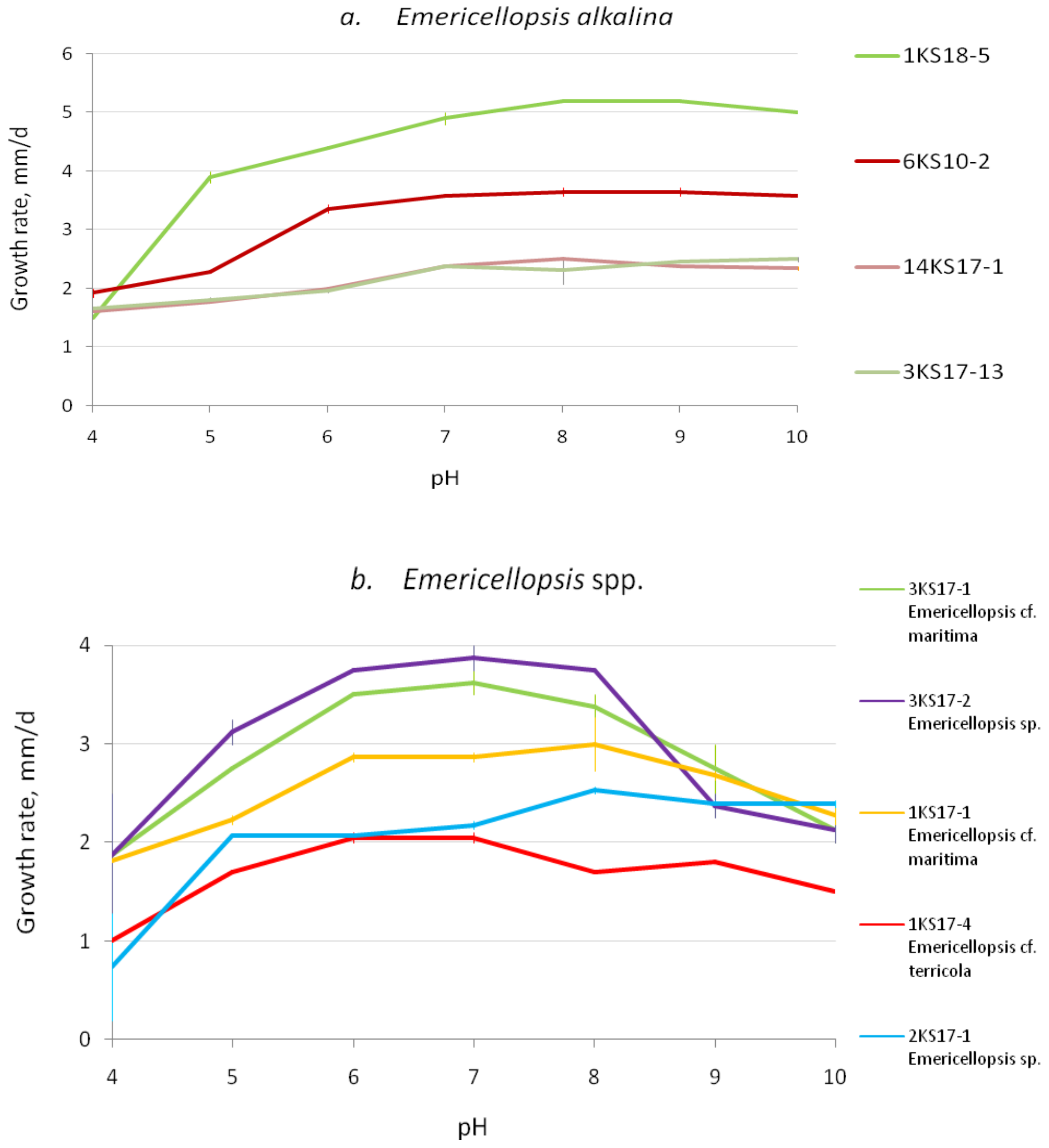

Figure S1. Growth patterns of the *Emericellopsis* strains at pH 4 through 10. Inoculation was done with a mycelium plug (1 mm) placed in the centre of a plate. The diameters of the colonies were measured every 2–4 days until they reached the edge of the plate. Growth rate was calculated as mm/day during linear growth of the fungus. Results presented on the graphs include population standard deviation. A. All *E. alkalina* strains showed good growth ability from neutral with pH 7 to alkaline medium with pH 10, had an optimum growth at pH 9. B. Other *Emericellopsis* strains showed different growth ability and the pH preferences.

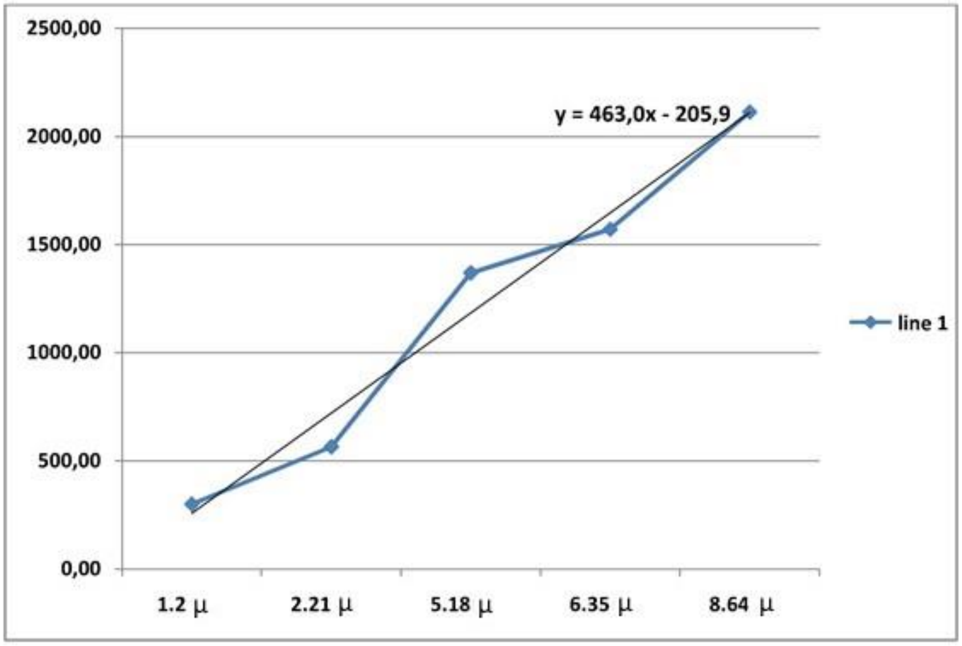

Figure S2. Emericellipsin A HPLC calibration

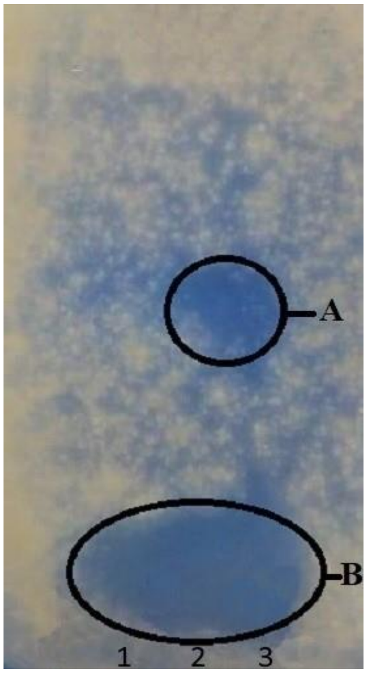

Figure S3. Bioautographic assay. Test culture is *A. niger* INA 00760, the elution system consist of CHCl3 (chloroform): MeOH (metanol) 3:1 respectively. The zones of growth inhibition are marked with black ovals. Black numbers 1, 2 and 3 are the concentrations of Emericellipsin A solutions 0,25 mg/ml; 0,5 mg/ml and 1 mg/ml respectively. A and B are two zones active against strain *A. niger* INA 00760.
